# Prevalence and epidemiological investigation of mgrB-dependent colistin resistance in extensively drug resistant *Klebsiella pneumoniae* in Iran

**DOI:** 10.1038/s41598-023-37845-z

**Published:** 2023-07-01

**Authors:** Abed Zahedi Bialvaei, Parisa Eslami, Leila Ganji, Alireza Dolatyar Dehkharghani, Farhad Asgari, Hossein Koupahi, Hamid Reza Barzegarian Pashacolaei, Mohammad Rahbar

**Affiliations:** 1 Microbial Biotechnology Research Center, Iran University of Medical Sciences, Tehran, Iran; 2 Department of Microbiology, Milad Hospital, Tehran, Iran; 3 Department of Microbiology, Ministry of Health & Medical Education, Iranian Reference Health Laboratories Research Center, Tehran, Iran; 4 Department of Microbiology, Islamic Azad University, Varamin-Pishva Branch, Varamin, Iran; 5 Department of Laboratory, Emam Hossein Hospital, Tehran, Iran

**Keywords:** Antimicrobials, Applied microbiology, Microbial genetics

## Abstract

Carbapenemases-producing *K. pneumoniae* are challenging antimicrobial therapy of hospitalised patients, which is further complicated by colistin resistance. The aim of this study was to investigate the molecular epidemiological insights into carbapenemases-producing and colistin-resistant clinical *K. pneumoniae*A total of 162 colistin resistant clinical strains of *K. pneumoniae* were collected during 2017–2019. Antimicrobial susceptibility and the colistin minimum inhibitory concentration were determined. Using PCR assay, the prevalence of resistance-associated genes including *bla*_KPC_, *bla*_IMP_, *bla*_VIM_, *bla*_OXA-_48, *bla*NDM_-1_ and *mcr*-1 to -9 was examined. Additionally, a PCR assay was used to examine the *mgrB* gene in colistin-resistant bacteria. 94.4% of the tested strains were resistant to imipenem and 96.3% were resistant to meropenem. Colistin resistance (MIC > 4 µg/L) was observed in 161 isolates (99.4%) by Colistin Broth Disk Elution method. The KPC enzyme was the most common carbapenemase and was identified in 95 strains (58.6%), followed by the IMP, VIM and OXA-48 detected in 47 (29%), 23 (14.2%) and 12 (7.4%) isolates, respectively. However, no NDM-1 gene was detected. Additionally, none of the studied isolates harbored mcr variants, while *mgrB* gene was observed in 152 (92.6%) isolates. Colistin resistance of *K. pneumoniae* isolates may be associated with *mgrB* gene mutation. To stop the spread of resistant *K. pneumoniae*, surveillance must be improved, infection prevention protocols must be followed, and antibiotic stewardship must be practised.

## Introduction

Multidrug-resistant Gram-negative bacteria (MDR-GNB), such as *Klebsiella pneumoniae*, are a serious public health concern, particularly infections caused by strains that produce carbapenemase and are only susceptible to a limited number of antimicrobials^1,2^. Polymyxin antibiotics have historically been used as a last resort to treat infections brought on by *Enterobacteriaceae* that are resistant to the antibiotic carbapenem.

Polymyxin antibiotics, including colistin (also known as polymyxin E) are cationic antimicrobial peptides that bind to the lipid A phosphate moiety of bacterial lipopolysaccharide (LPS), resulting in leakage of intracellular components from the cell membrane^3^. However, the emergence of colistin resistance in GNB has been reported in several countries, with resistance mediated via genetic variations represented by chromosomal mutations in genes involved in lipopolysaccharide synthesis, namely *phoP*/*phoQ*, *pmrA*/*pmrB* or *crrA*/*crrB* as well as on the *mgrB* regulatory gene^4,5^. *K. pneumoniae*'s acquired colistin resistance has been linked to the inactivation of the *mgrB* gene, with several genetic events causing changes to these genes in both human and animal isolates^6^. These mutations lead to overexpression of the genes and an increased synthesis of phospho- ethanolamine (pEtN) and 4-amino-4-deoxy-l-arabinose (LAra4N)^7^.

In 2016, Yi-Yun Liu et al. discovered a plasmid-mediated colistin resistance gene (*mcr*-1) encoding a lipid A phosphoethanolamine transferase that confers resistance to colistin by transferring pEtN to lipid A^8^. Colistin-resistant *Enterobacteriaceae*, particularly *K. pneumoniae*, that contain the *mcr*-1 gene have been reported from humans, animals used for food production, and the environment worldwide, raising the possibility of horizontal transmission of colistin resistance^9^. This has raised concerns about the potential emergence of pandrug resistance in Enterobacterales. As a result, it is important to continuously and precisely examine how the *mcr* genes emerged and propagated across bacteria. A systematic review and meta-analysis on the prevalence of colistin resistance of *K. pneumoniae* isolates in Iran revealed that the pooled prevalence of colistin resistance in clinical isolates was 6.9%^10^. However, the rate of *K. pneumoniae* carbapenem resistance was more than 73% in different studies^11,12^.

The development of reliable techniques for the detection of polymyxin resistance, with low cost and feasibility are necessary. Simner and colleagues described the Colistin Broth Disk Elution (CBDE), which uses colistin disks as a source of these antibiotics^13^. This study’s objective was to examine the molecular mechanisms of colistin and carbapenem resistance among a collection of extensively drug resistant *K. pneumoniae* collected from clinical specimens in Tehran, Iran. Also, a comparison was made in susceptibility of *K. pneumoniae* isolates to colistin using Disk diffusion, Chrome Agar, E-test, and CBDE.

## Materials and methods

### Bacterial strains

From June 2017 to March 2019, a total of 162 non-duplicate strains of *K. pneumoniae* were isolated from clinical samples of inpatients and outpatients at Milad Hospital in Tehran, and they were resistant to the colistin in the initial screening. The disc diffusion method and selective agar medium CHROMagar COL-APSE (Paris, France) were used to detect resistance to colistin and finally colistin resistant isolates that were confirmed by both methods were included in the study.

### Antimicrobial susceptibility testing

Antimicrobial susceptibility testing was performed on Muller– Hinton agar plates using the standard disk diffusion method according to the Clinical and Laboratory Standards Institute (CLSI, 2022) guidelines^14^. A total of 10 antibiotics including cefotaxime (CTX: 30 μg), ceftazidime (CAZ: 30 μg), piperacillin/tazobactam (TZP: 100/10 μg), amikacin (AN: 30 μg), gentamicin (GM: 10 μg), fosfomycin (FOX: 200 μg), imipenem (IMP: 10 μg), meropenem (MERO: 10 μg), ciprofloxacin (CIP: 5 μg), and trimethoprim/sulfamethoxazole (SXT: 1.25/23.75 μg) were investigated. *Escherichia coli* ATCC 25,922 was used as a quality control strain.

Minimum inhibitory concentrations (MICs) of colistin were determined using E-test strips (bioMérieux, Craponne, France) and CBDE^13^. Isolates with an MIC > 4 µg/L by CBDE were categorized as resistant.

### Molecular analysis of carbapenem and colistin resistance-associated genes

DNA extraction was performed commercial High pure PCR template preparation kit (Roche Molecular Biochemicals, IN, USA) according to the manufacturer’s instructions. Carbapenemases-encoding genes such as (blaKPC, blaIMP, blaVIM, blaOXA-48 and blaNDM-1) were detected by singleplex PCR^15–19^. Singleplex PCRs were also used to test the colistin-resistant isolates for the presence of mcr-1–9 according to previous published papers^20–24^. In addition, using specific primers that targeted the *mgrB* coding sequence and several flanking areas, PCR analysis of *mgrB* was carried out^25^. The sequence of primers used in this study, which were selected based on common variants, is shown in Table 1. Positive strains for blaNDM, blaVIM, blaKPC and blaOXA-48 that confirmed by sequencing method were used as positive control and *E. coli* ATCC 25922 was utilized as negative control in the PCR assays.

**Table 1:** Primers used in this study.

Primers	Sequences	Size (bp)	References
KPC	ATGTCACTGTATCGCCGTCT TTTTCAGAGCCTTACTGCCC	893	^15^
NDM-1	GGTTTGGCGATCTGGTTTTC CGGAATGGCTCATCACGATC	621	^16^
OXA-48	TTGGTGGCATCGATTATCGG GAGCACTTCTTTTGTGATGGC	743	^17^
IMP	CCAAACYACTASGTTATC GAATAGRRTGGCTTAAYTCTC	188	^18^
VIM	GTTTGGTCGCATATCGCAAC AATGCGCAGCACCAGGATAG	382	^19^
Mcr-1	AGTCCGTTTGTTCTTGTGGC AGATCCTTGGTCTCGGCTTG	320	^20^
Mcr-2	AGCCGAGTCTAAGGACTTGATGAATTTG GCGGTATCGACATCATAGTCATCTTG	576	^21^
Mcr-3	AAATAAAAATTGTTCCGCTTATG AATGGAGATCCCCGTTTTT	929	^20^
Mcr-4	AATTGTCGTGGGAAAAGCCGC CTGCTGACTGGGCTATTACCGTCAT	1062	^22^
Mcr-5	GTGAAACAGGTGATCGTGACTTACCG CGTGCTTTACACCGATCATGTGCT	271	^23^
Mcr-6	ACTGACCAAGCCGAGTCTAAG GCATCACGGGATTGACATAGC	259	^24^
Mcr-7	GCGACCTCCTACCTGAATG CCCTTTGGCGACGACTTTG	345	^24^
Mcr-8	TTGTCGTCGTGGGCGAAAC CTGTCGCAAGTTGGGCTAAAG	514	^24^
Mcr-9	CGGCGAACTACGCTTACAG CGCACAGTTTCGGGTTATCAC	465	^24^
mgrB	AAGGCGTTCATTCTACCACC TTAAGAAGGCCGTGCTATCC	253	^25^

### Genomic sequencing

Draft genome sequences were created using genomic sequencing on an Oxford Nanopore GridION in National Reference Laboratory.

### Statistical analysis

Data analysis was performed using IBM SPSS Statistics v.22.0 (IBM Corp., Armonk, NY). The frequency of isolates between groups was compared by χ2 test. A P-value of ≤ 0.05 was considered statistically significant.

## Results

### Epidemiology of colistin-resistant *K. pneumoniae* isolates

During the study periods, 162 colistin resistant strains of *K. pneumoniae* belonging to 94 (58%) men and 68 (42%) women were studied. The average age of the patients was 67.25 ± 1.2 (between 19 and 98 years) and most of the studied patients 103 (63.6%) were older than 65 years. Out of 162 patients, 155 were inpatients and only 7 were outpatients. The largest number of studied samples included trachea (41, 25.3%), urine (37, 22.8%) and sputum (35, 21.6%) samples, more than half of which (95, 58.6%) were received from the intensive care unit (ICU). The demographic characteristics of patients, sample types and distribution of study isolates among the different hospital wards are shown in Table 2.

**Table 2:** Sources of strains isolation.

Characteristics	N (%)
Gender, N (%)	
Male	94 (58)
Female	68 (42)
Age, N (%)	
19–24	1 (0.6)
25–34	5 (3.1)
35–44	9 (5.6)
45–54	14 (8.6)
55–64	30 (18.5)
65 +	103 (63.6)
Specimen type (%)	
Wound	17 (10.5)
Sputum	35 (21.6)
Urine	37 (22.8)
Trachea	41 (25.3)
Blood	16 (9.9)
BAL	1 (0.6)
Pleural	5 (3.1)
Ascitic fluid	3 (1.9)
Abscess	3 (1.9)
Urinary catheter	4 (2.4)
Ward (%)	
Internal	29 (17.9)
I.C.U	95 (58.6)
Kidney transplantation	6 (3.7)
Emergency	6 (3.7)
Lung	6 (3.7)
Surgery	9 (5.6)
C.C.U	3 (1.9)
Dialysis	1 (0.6)
Outpatients	7 (4.3)
Total	162 (100)

### Antimicrobial susceptibility testing

**Figure 1:**
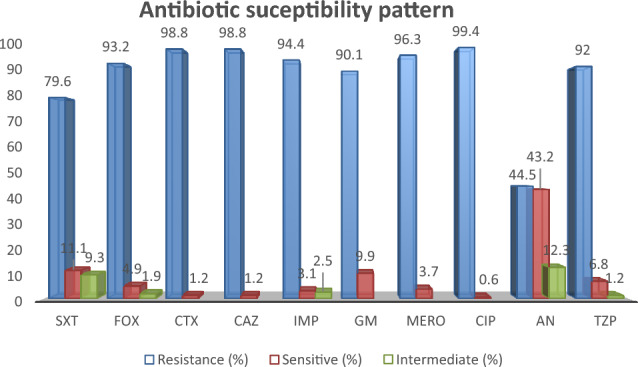
Results of Antimicrobial susceptibility testing for different antibiotics in colistin resistant *K. pneumoniae* isolates. *SXT* Trimethoprim/Sulfamethoxazole, *FOX* Fosfomycin, *CTX* cefotaxime, *CAZ* Ceftazidime, *IMP* Imipenem, *GM* Gentamicin, *MERO* Meropenem, *CIP* Ciprofloxacin, *AN* Amikacin, *TZP* Piperacillin-Tazobactam.

79.6% of the tested strains were resistant to cotrimoxazole, and except for amikacin, resistance to other antibiotics was observed above 90% (Fig. 1). Out of 162 K*. pneumoniae* isolates, 161 (99.3%) were resistant to ciprofloxacin. The resistance rate of the third generation of cephalosporins including ceftriaxone and ceftazidime was 98.8%. Resistance to carbapenems, which are used as alternative antibiotics in the treatment of strains resistant to cephalosporins, was high, so that 94.4% of the tested strains were resistant to imipenem and 96.3% were resistant to meropenem. From aminoglycoside antibiotics, gentamycin and amikacin were tested, and the resistance to gentamycin was about 90%, while the resistance to amikacin was about 44%, and 12% of the strains had intermediate resistance to this antibiotic. Resistance of *K. pneumoniae* strains to tazobactam-piperacillin was 92%. Colistin resistance (MIC > 4 µg/L) was observed in 161 isolates (99.4%) by CBDE (Table 3). To compare the antimicrobial sensitivity test using chrome agar, E-test and CBDE methods in colistin resistant *K. pneumoniae* isolates, 95.6% of the methods used in this study overlapped to detect colistin sensitivity. Meanwhile, a big difference was observed with the disc diffusion method. 50 (30.8%) of the isolates that showed resistance to colistin by other studied methods, showed a sensitive phenotype by disc diffusion method.

**Table 3:** Comparison of colistin resistance results obtained with different methods in *K. pneumoniae* isolates.

Test	Resistance (%)	Sensitive (%)
CBDE	161 (99.4)	1 (0.6)
Chrome Agar	154 (95.1)	8 (4.9)
MIC (E-test)	160 (98.8)	2 (1.2)
Disk diffusion	105 (64.8)	57 (35.2)
Total	162 (100)	

### Molecular analysis of carbapenem and colistin resistance-associated genes

The gene encoding the KPC enzyme was the most common and was identified in 95 strains (58.6%), followed by IMP and VIM in 47 (29%) and 23 (14.2%) strains, respectively. The gene encoding the OXA-48 enzyme was found in 12 strains (7.4%), while the NDM-1 gene was not detected.

PCR screening for plasmid-mediated colistin resistance genes (*mcr*-1 to *mcr*-9) were examined on tested strains. According to the findings, no positive *mcr* isolates were found. Therefore, all the strains were examined for the presence of the *mgrB* gene, and 152 (92.6%) strains harboured the gene. Genomic sequencing of strains harboured *mgrB* gene revealed that only the conversion of codon 39 from TCA to TGA was present in two isolates, since the TGA codon is a stop codon, which causes the termination of transcription and the loss of protein function (Accession number KF852760: m colistin-susceptible *K. pneumoniae* strain; coverage:53%).

## Discussion

The global increase in multidrug-resistant *K. pneumoniae* strains has increased the use of colistin to treat these infections, resulting in the emergence of colistin resistance worldwide^26,27^. The important concern that must be considered in the management of nosocomial infections caused by *K. pneumoniae* are periodic surveillance to identify the resistant strains for optimizing available infection control policies and treatment options in different parts of the hospitals^28^. This study investigated the molecular mechanisms of colistin and carbapenem resistance among a collection of extensively drug resistant *K. pneumoniae* clinical isolates in Tehran, Iran. Also, different phenotypic methods including disc diffusion, E-test, chrome agar and CBDE were compared to determine the susceptibility of *K. pneumoniae* isolates to colistin.

In the comparison between the phenotypic methods, the highest number of colistin-resistant strains was identified with the CBDE method. So that out of 162 tested strains, except for one strain, the other isolates were resistant to colistin by the CBDE method, then the E-test and chrome agar methods showed the highest resistance (95.6% overlap to detect colistin resistance), and finally, the disc diffusion method showed the lowest resistance. Some studies have compared agar dilution, E-test and disk diffusion methods to measure colistin resistance in Gram-negative bacilli^29,30^. According to CLSI and EUCAST guidelines, they confirmed that the use of diffusion-based methods for antimicrobial susceptibility testing against colistin was unreliable, which is due to the large size of the colistin, prevents its uniform diffusion in agar-containing media^14^. Despite this recommendation, the results of this study showed that the disk diffusion method performed with both types of commercial disks containing colistin was able to successfully differentiate resistant strains. According to this result, it seems that revisions should be made about the diagnostic value of the disc diffusion method to determine the susceptibility to colistin and this test should not be completely abandoned. Because disc diffusion method is able to identify at least strains with high level of resistance to colistin and can be an important tool in the direction of rapid screening of resistant strains with high resistance level. CBDE is a simple and low-cost phenotypic method to test antimicrobial sensitivity against colistin in Gram-negative bacilli, including *Enterobacteriaceae*, which has been approved by CLSI and EUCAST. Studies have shown that CBDE method was comparable with the broth microdilution method as a reference method with a 100% correlation^13,31^.

The colistin-resistant rate in Iran were reported about 11.6%^32^. Nevertheless, data from reports of neighboring countries showed the resistance to colistin are ranging from 0 to 31.7%^33^. As expected for colistin-resistant isolates in our study, most were also resistant to other clinically useful antimicrobial agents. In the present study, 96% of isolates showed phenotypic resistance to meropenem and/or imipenem, which similar to many other studies, 95 of them harboured *bla*_KPC_ gene^34^. We found that none of the colistin-resistant *K. pneumoniae* isolates had plasmid-encoded *mcr*-genes, suggesting that the resistance is mediated by chromosomally encoded mechanisms. MCR-1 is still extremely uncommon in clinical isolates worldwide^35^. In previous investigations, *mcr*-1 prevalence in *Enterobacteriaceae* was reported to range between 0.1 and 1%^36,37^. The literature shows the prevalence of the *mcr*-1 gene was lower in *Enterobacteriaceae* strains isolated from human sources than in strains isolated from animal and food samples^38^. This assumes that their reservoir is at least in animals and the environment, following the important use of colistin in animal production and in general agriculture. In Iran, the massive use of colistin in clinical practice following the spread of carbapenemase-producing *Enterobacteriaceae* has led to the selection of multidrug-resistant bacteria in hospital settings^39^.

Interestingly, the results of the current study showed that *mcr*-1- negative *K. pneumoniae* isolates had high level of colistin resistance. Consistent with this findings, previous studies reported that *K. pneumoniae* with chromosomal mutations in *mgrB* exhibited a high level of colistin resistance^40,41^. The mechanisms of colistin resistance other than those attributed to the *mcr*-1 gene in the *K. pneumoniae* isolates in this study are being further studied to define the precise molecular mechanism of resistance. Of 162 colistin resistant *K. pneumoniae* isolates, 150 (92.6%) isolates had *mgrB*. Critical changes in *mgrB*, such as disruption of the promoter or coding sequence, are thought to cause the gene to be silenced or lead to the generation of shortened *mgrB*^40^. In reality, PhoP/PhoQ activation follows *mgrB* inactivation by any of these occurrences, which in turn activates the PmrA response regulator responsible for modification of the lipopolysaccharide polymyxin target^25^. So mentioned mutation in the present study may causes the termination of transcription and the loss of protein function. Avgoulea et al. reported that insertional inactivation of the *mgrB* gene conferred resistance to colistin in all isolates tested^42^.

There were some limitations related to the present study. Firstly, it should be noted that this study was performed using extracted DNA samples from *K. pneumoniae* that were selected based on the results of initial screening for colistin resistance. The lack of strains carrying *mcr* genes except mcr-1 as a positive control is another limitation. From an epidemiological standpoint, the monocentric nature of the study is a significant limitation.

## Conclusion

Our findings indicate that the colistin resistance of *K. pneumoniae* isolates may be associated with *mgrB* gene mutation. These data provide added insight into the mechanism of colistin. Colistin resistance developed with a number of new mutations among highly resistant populations, limiting the availability of further antimicrobial medicines and resulting in pandrug resistance. The prevalence of resistance to carbapenems and colistin in Iran should be surveyed and new therapeutic strategies including old drugs should be evaluated and used in Iran.

## Data Availability

All data generated or analyzed during this study are included in this published article and Supplementary Information file [and its tables and figures].

## Code availability

The code is available from the corresponding author upon request.

## References

[1] Jamali S, Tavakoly T, Mojtahedi A, Shenagari M (2020). The phylogenetic relatedness of bla NDM-1 harboring extended-spectrum β-lactamase producing uropathogenic *Escherichia coli* and *Klebsiella pneumoniae* in the North of Iran. Infect. Drug Resist..

[2] Pirzaman AN, Mojtahedi A (2019). Investigation of antibiotic resistance and the presence of integron genes among ESBL producing Klebsiella isolates. Meta Gene.

[3] Yap PS-X, Cheng W-H, Chang S-K, Lim S-HE, Lai K-S (2022). MgrB mutations and altered cell permeability in colistin resistance in *Klebsiella pneumoniae*. Cells.

[4] Poirel L, Jayol A, Bontron S, Villegas M-V, Ozdamar M, Türkoglu S (2015). The mgrB gene as a key target for acquired resistance to colistin in *Klebsiella pneumoniae*. J. Antimicrob. Chemother..

[5] Cheong HS, Kim SY, Wi YM, Peck KR, Ko KS (2019). Colistin heteroresistance in *Klebsiella pneumoniae* isolates and diverse mutations of PmrAB and PhoPQ in resistant subpopulations. J. Clin. Med..

[6] Yusof NY, Norazzman NII, Hakim SNWA, Azlan MM, Anthony AA, Mustafa FH (2022). Prevalence of mutated colistin-resistant *Klebsiella pneumoniae*: A systematic review and meta-analysis. Trop. Med. Infect. Dis..

[7] Gerson S, Betts JW, Lucaßen K, Nodari CS, Wille J, Josten M (2019). Investigation of novel pmrB and eptA mutations in isogenic *Acinetobacter baumannii* isolates associated with colistin resistance and increased virulence in vivo. Antimicrob. Agents Chemother..

[8] Liu Y-Y, Wang Y, Walsh TR, Yi L-X, Zhang R, Spencer J (2016). Emergence of plasmid-mediated colistin resistance mechanism MCR-1 in animals and human beings in China: A microbiological and molecular biological study. Lancet. Infect. Dis..

[9] Poirel L, Jayol A, Nordmann P (2017). Polymyxins: Antibacterial activity, susceptibility testing, and resistance mechanisms encoded by plasmids or chromosomes. Clin. Microbiol. Rev..

[10] Narimisa N, Goodarzi F, Bavari S (2022). Prevalence of colistin resistance of *Klebsiella pneumoniae* isolates in Iran: A systematic review and meta-analysis. Ann. Clin. Microbiol. Antimicrob..

[11] Sharahi JY, Hashemi A, Ardebili A, Davoudabadi S (2021). Molecular characteristics of antibiotic-resistant *Escherichia coli* and *Klebsiella pneumoniae* strains isolated from hospitalized patients in Tehran, Iran. Ann. Clin. Microbiol. Antimicrob..

[12] Mokhtari M, Mojtahedi A, Mahdieh N, Jafari A, Arya MJ (2022). High prevalence of blaOXA-48 and blaNDM-producing carbapenem-resistant *Klebsiella pneumoniae* isolated from clinical samples in Shahid Rajaei Hospital in Tehran, Iran. Jundishapur J. Microbiol..

[13] Simner PJ, Bergman Y, Trejo M, Roberts AA, Marayan R, Tekle T (2019). Two-site evaluation of the colistin broth disk elution test to determine colistin in vitro activity against Gram-negative bacilli. J. Clin. Microbiol..

[14] Clinical and Laboratory Standards Institute (2022). Performance Standards for Antimicrobial Susceptibility Testing, M100.

[15] Handal R, Qunibi L, Sahouri I, Juhari M, Dawodi R, Marzouqa H (2017). Characterization of carbapenem-resistant *Acinetobacter baumannii* strains isolated from hospitalized patients in Palestine. Int. J. Microbiol..

[16] Nordmann P, Poirel L, Carrër A, Toleman MA, Walsh TR (2011). How to detect NDM-1 producers. J. Clin. Microbiol..

[17] Brink AJ, Coetzee J, Corcoran C, Clay CG, Hari-Makkan D, Jacobson RK (2013). Emergence of OXA-48 and OXA-181 carbapenemases among Enterobacteriaceae in South Africa and evidence of in vivo selection of colistin resistance as a consequence of selective decontamination of the gastrointestinal tract. J. Clin. Microbiol..

[18] Vasconcelos NG, Queiroz JHFDS, Silva KED, Vasconcelos PCDP, Croda J, Simionatto S (2020). Synergistic effects of *Cinnamomum cassia* L. essential oil in combination with polymyxin B against carbapenemase-producing *Klebsiella pneumoniae* and *Serratia marcescens*. PLoS ONE.

[19] Fallah F, Borhan RS, Hashemi A (2013). Detection of bla (IMP) and bla (VIM) metallo-β-lactamases genes among *Pseudomonas aeruginosa* strains. Int. J. Burns Trauma.

[20] Rebelo AR, Bortolaia V, Kjeldgaard JS, Pedersen SK, Leekitcharoenphon P, Hansen IM (2018). Multiplex PCR for detection of plasmid-mediated colistin resistance determinants, mcr-1, mcr-2, mcr-3, mcr-4 and mcr-5 for surveillance purposes. Euro Surveill..

[21] Yassin AK, Zhang J, Wang J, Chen L, Kelly P, Butaye P (2017). Identification and characterization of mcr mediated colistin resistance in extraintestinal *Escherichia coli* from poultry and livestock in China. FEMS Microbiol. Lett..

[22] Chen L, Zhang J, Wang J, Butaye P, Kelly P, Li M (2018). Newly identified colistin resistance genes, mcr-4 and mcr-5, from upper and lower alimentary tract of pigs and poultry in China. PLoS ONE.

[23] Zhang J, Chen L, Wang J, Butaye P, Huang K, Qiu H (2018). Molecular detection of colistin resistance genes (mcr-1 to mcr-5) in human vaginal swabs. BMC. Res. Notes.

[24] Gorecki A, Musialowski M, Wolacewicz M, Decewicz P, Ferreira C, Vejmelkova D (2022). Development and validation of novel PCR primers for identification of plasmid-mediated colistin resistance (mcr) genes in various environmental settings. J. Hazard. Mater..

[25] Cannatelli A, D'Andrea MM, Giani T, Di Pilato V, Arena F, Ambretti S (2013). In vivo emergence of colistin resistance in *Klebsiella pneumoniae* producing KPC-type carbapenemases mediated by insertional inactivation of the PhoQ/PhoP mgrB regulator. Antimicrob. Agents Chemother..

[26] Wang Y, Liu F, Hu Y, Zhang G, Zhu B, Gao GF (2020). Detection of mobile colistin resistance gene mcr-9 in carbapenem-resistant *Klebsiella pneumoniae* strains of human origin in Europe. J. Infect..

[27] Zahedi Bialvaei A, Dolatyar Dehkharghani A, Asgari F, Shamloo F, Eslami P, Rahbar M (2021). Modified CIM test as a useful tool to detect carbapenemase activity among extensively drug-resistant *Klebsiella pneumoniae*, *Escherichia coli* and *Acinetobacter baumannii*. Ann. Microbiol..

[28] Ghaffarian F, Hedayati M, Ebrahim-Saraie HS, Roushan ZA, Mojtahedi A (2018). Molecular epidemiology of ESBL-producing *Klebsiella pneumoniae* isolates in intensive care units of a tertiary care hospital, North of Iran. Cell Mol. Biol. (Noisy-le-grand).

[29] Galani I, Kontopidou F, Souli M, Rekatsina P-D, Koratzanis E, Deliolanis J (2008). Colistin susceptibility testing by Etest and disk diffusion methods. Int. J. Antimicrob. Agents.

[30] Haeili M, Kafshdouz M, Pishnian Z, Feizabadi MM, Martinez-Martinez L (2019). Comparison of susceptibility testing methods for determining the activity of colistin against Gram-negative bacilli of clinical origin. J. Med. Microbiol..

[31] Humphries RM, Green DA, Schuetz AN, Bergman Y, Lewis S, Yee R (2019). Multicenter evaluation of colistin broth disk elution and colistin agar test: A report from the Clinical and Laboratory Standards Institute. J. Clin. Microbiol..

[32] Aghapour Z, Hasani A, Aghazadeh M, Rezaee MA, Ganbarov K, Pourlak T (2019). Genes involved in colistin resistance of gram-negative isolates in the northwest of Iran. Gene Rep..

[33] Bialvaei AZ, Samadi KH (2015). Colistin, mechanisms and prevalence of resistance. Curr. Med. Res. Opin..

[34] Hu Y, Liu C, Wang Q, Zeng Y, Sun Q, Shu L (2021). Emergence and expansion of a carbapenem-resistant *Pseudomonas aeruginosa* clone are associated with plasmid-borne bla KPC-2 and virulence-related genes. Msystems.

[35] Aris P, Robatjazi S, Nikkhahi F, Marashi SMA (2020). Molecular mechanisms and prevalence of colistin resistance of *Klebsiella pneumoniae* in the Middle East region: A review over the last 5 years. J. Glob. Antimicrob. Res..

[36] Castanheira M, Griffin MA, Deshpande LM, Mendes RE, Jones RN, Flamm RK (2016). Detection of mcr-1 among *Escherichia coli* clinical isolates collected worldwide as part of the SENTRY Antimicrobial Surveillance Program in 2014 and 2015. Antimicrob. Agents Chemother..

[37] Quan J, Li X, Chen Y, Jiang Y, Zhou Z, Zhang H (2017). Prevalence of mcr-1 in *Escherichia coli* and *Klebsiella pneumoniae* recovered from bloodstream infections in China: A multicentre longitudinal study. Lancet Infect..

[38] Elbediwi M, Li Y, Paudyal N, Pan H, Li X, Xie S (2019). Global burden of colistin-resistant bacteria: Mobilized colistin resistance genes study (1980–2018). Microorganisms.

[39] Fayyaz M, Zafar H, Mirza IA, Meral U, Hussain A, Khalil H (2022). In vitro susceptibility of colistin against multidrug resistant *Klebsiella pneumoniae* clinical isolates. Pak. Armed Forces Med. J..

[40] Haeili M, Javani A, Moradi J, Jafari Z, Feizabadi MM, Babaei E (2017). MgrB alterations mediate colistin resistance in *Klebsiella pneumoniae* isolates from Iran. Front. Microbiol..

[41] Jayol A, Nordmann P, Lehours P, Poirel L, Dubois V (2018). Comparison of methods for detection of plasmid-mediated and chromosomally encoded colistin resistance in Enterobacteriaceae. Clin. Microbiol. Infect..

[42] Avgoulea K, Di Pilato V, Zarkotou O, Sennati S, Politi L, Cannatelli A (2018). Characterization of extensively drug-resistant or pandrug-resistant sequence type 147 and 101 OXA-48-producing *Klebsiella pneumoniae* causing bloodstream infections in patients in an intensive care unit. Antimicrob. Agents Chemother..

